# Artificial Cell Encapsulation for Biomaterials and Tissue Bio-Nanoengineering: History, Achievements, Limitations, and Future Work for Potential Clinical Applications and Transplantation

**DOI:** 10.3390/jfb12040068

**Published:** 2021-11-30

**Authors:** Armin Mooranian, Melissa Jones, Corina Mihaela Ionescu, Daniel Walker, Susbin Raj Wagle, Bozica Kovacevic, Jacqueline Chester, Thomas Foster, Edan Johnston, Jafri Kuthubutheen, Daniel Brown, Momir Mikov, Hani Al-Salami

**Affiliations:** 1 The Biotechnology and Drug Development Research Laboratory, Curtin Medical School & Curtin Health Innovation Research Institute, Curtin University, Bentley, Perth, WA 6102, Australia; A.Mooranian@curtin.edu.au (A.M.); melissa.a.jones@postgrad.curtin.edu.au (M.J.); c.ionescu@postgrad.curtin.edu.au (C.M.I.); daniel.walker1@postgrad.curtin.edu.au (D.W.); susbinraj.wagle@postgrad.curtin.edu.au (S.R.W.); bozica.kovacevic@postgrad.curtin.edu.au (B.K.); j.chester@student.curtin.edu.au (J.C.); thomas.p.foster@student.curtin.edu.au (T.F.); edan.johnston@student.curtin.edu.au (E.J.); 2 Hearing Therapeutics, Ear Science Institute Australia, Queen Elizabeth II Medical Centre, Nedlands, Perth, WA 6009, Australia; 3 Fiona Stanley Hospital, Murdoch, WA 6150, Australia; Jafri.Kuthubutheen@health.wa.gov.au; 4 Curtin Medical School, Curtin Health Innovation Research Institute, Curtin University, Perth, WA 6102, Australia; daniel.brown2@curtin.edu.au; 5 Department of Pharmacology, Toxicology and Clinical Pharmacology, Faculty of Medicine, University of Novi Sad, Hajduk Veljkova 3, 21101 Novi Sad, Serbia; momir.mikov@mf.uns.ac.rs

**Keywords:** artificial cells, diabetes mellitus, encapsulation, insulinoma cell lines, islet transplantation

## Abstract

Pancreatic β-cell loss and failure with subsequent deficiency of insulin production is the hallmark of type 1 diabetes (T1D) and late-stage type 2 diabetes (T2D). Despite the availability of parental insulin, serious complications of both types are profound and endemic. One approach to therapy and a potential cure is the immunoisolation of β cells via artificial cell microencapsulation (ACM), with ongoing promising results in human and animal studies that do not depend on immunosuppressive regimens. However, significant challenges remain in the formulation and delivery platforms and potential immunogenicity issues. Additionally, the level of impact on key metabolic and disease biomarkers and long-term benefits from human and animal studies stemming from the encapsulation and delivery of these cells is a subject of continuing debate. The purpose of this review is to summarise key advances in this field of islet transplantation using ACM and to explore future strategies, limitations, and hurdles as well as upcoming developments utilising bioengineering and current clinical trials.

## 1. Introduction

One of the fastest growing metabolic diseases of the twenty first century is diabetes mellitus (DM), of which there are two primary classifications, historically differentiated based upon the degree of insulin production, with no production in type 1 diabetes (T1D), to moderate production, classified as type 2 diabetes (T2D) [1,2,3]. These two classical sub-types of DM are not as distinct and mutually exclusive as their traditional definitions seem to suggest. There are some similarities that deserve attention; including the fact that both are associated with bile acid disturbances, inflammation with tissue necrosis, and altered gastrointestinal microbial content [4,5]. In 2016, Schwartz et al., suggested that diabetes should instead be classified based upon a β-cell-centric model, to alleviate issues that the current classification presents in diagnosis and treatment, with other researchers also suggesting other, improved classifications, to assist with overlapping treatment models [6,7]. Further adding to the crossover between types is the fact that many T2D patients eventually require exogenous insulin due to significant β-cell damage that occurs with longstanding T2D. This is often a side effect of chronic drug therapy, or when such drugs have failed to substantiate glucose control; particularly with the use of sulphonylureas such as Gliclazide [8,9,10]. 

Based upon decades of research, it is clear that there is currently no adequate stand-alone therapy that is sufficient for the treatment of all aspects of diabetes; with current regimens typically associated with side effects, treatment failures and patient compliance issues [1,11,12]. The hallmark of diabetic disease types is pancreatic islet dysfunction, warranting the use of exogenous insulin as replacement therapy (in T1D and also for late-stage T2D) [9,13]. However, this form of insulin therapy is associated with poor patient compliance, local injection site reactions, the risk of hypoglycaemia and associated complications including weight gain [14,15,16]. Ideally, the ultimate “cure” for diabetes mellitus would be the complete replacement of the diseased pancreas with a functional transplant [17,18]. Unfortunately, there are significant issues associated with organ transplantation such as the availability and quality of the pancreas from donors, and more so, the long-term use of toxic immunosuppressive regimens which are associated with significant adverse effects [19,20,21]. Pancreatic islet transplantation offers some advantages over whole-organ transplantation, including more availability to older patients due to the reduction in the invasive nature; but is severely limited by feasibility issues [20,21,22,23,24,25]. 

On the other hand, a rapidly expanding and very promising area is the encapsulation and immunoisolation of islet cells using appropriate polymers and excipients [26,27,28]. This technique procures the protection and proliferation of the cells in order to create a “bioartificial” pancreas with suitable biological characteristics and clinical utility [28]. Despite the milestones achieved in the past decade, there are challenges and limitations that need to be addressed and rectified before this form of pharmaceutical transplantation gains widespread acceptance [29,30]. 

**Figure 1:**
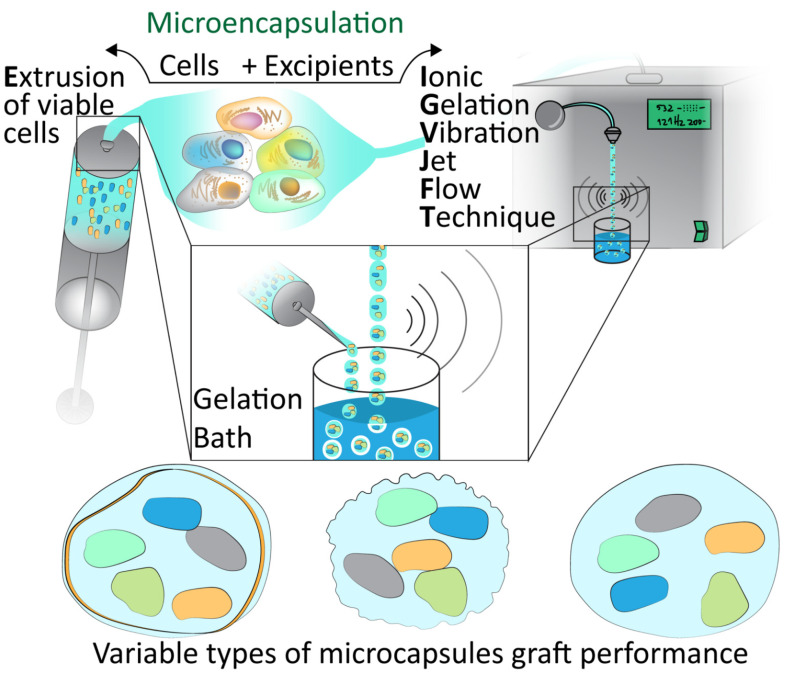
Microencapsulation techniques. Demonstration of the extrusion of viable cells and encapsulation with excipients via Ionic Gelation Jet Flow Techniques and gelation bath to form various types of microcapsules which must be assessed for graft performance.

**Figure 2:**
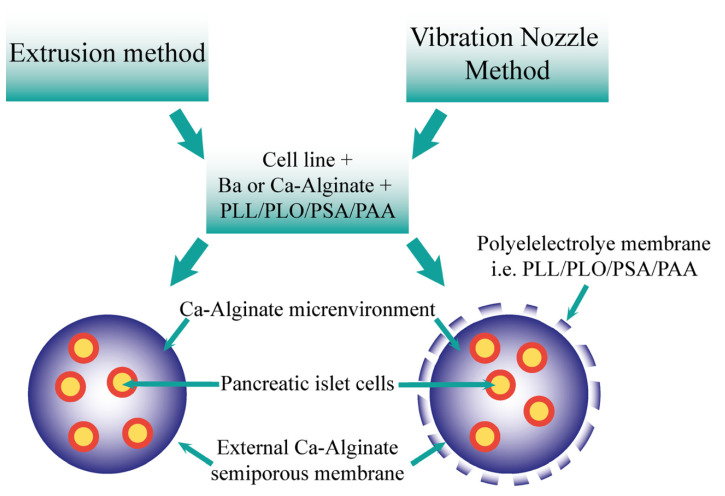
Comparison of the resultant microcapsules formed via the extrusion and vibration nozzle methods. The two methods may form microcapsules utilising the cells, ions, alginate, and polyelectrolytes. Both offer an ion–alginate environment for pancreatic islet cells, including semiporous membrane. The vibration nozzle method also produces a polyelectrolyte membrane.

Cell microencapsulation offers an alternative to the explored macroencapsulation in diabetes. The process of delivering insulinoma tissues in a membrane, an early suggestion of encapsulation, was first studied in 1933. Desai et al., developed biocapsules of the insulinoma cell lines, including from the RIN and βTC cell lines, including implantation in mice [31,32]. Systems such as these have drawbacks such as their ratio of surface area to volume, which would indicate diffusion and nutrient/waste exchange would be more limited. Such limitations may be overcome by cell microencapsulation, with demonstrations of such capsules seen in Figure 1 and Figure 2 [33].

Therefore, the aim of this review is principally three-fold. Firstly, this review will briefly outline diabetes mellitus with an overview, and explore the current treatments and limitations, including the existing transplantation options that are available as replacement therapy for the pancreas; secondly a comprehensive outline will be presented, highlighting various delivery platforms for islet cells and pharmaceutical technology involving multiple formulatory options and insulinoma cell lines which may be used for testing. Finally, the limitations of these techniques will be outlined, providing an insight into future directions on the horizon, including ongoing clinical trials relevant to the field of microencapsulation of pancreatic islet cells. 

## 2. Methodology

Review of the literature (restricted to English language only) was conducted using electronic databases such as PubMed^®^ ProQuest^®^, IPA^®^, Google scholar^®^, Science Direct^®^, Embase^®^ and SciFinder^®^ published after 2000. Keywords used included “diabetes mellitus”, “transplantation”, “β-cell line”, “artificial cell microencapsulation”, “pancreatic islets”, “bile acids” and “alginate.” If nonpancreatic cell lines were discussed, it was not included in the manuscript.

## 3. Diabetes Mellitus

### 3.1. Overview

The incidence of T1D, and more prominently, T2D, have significantly increased to become an escalating epidemiological problem of the current era. As of 2015, approximately 415 million people were classified as having T2D disease worldwide. Therefore, it is presumed that such a metabolic disorder will continue to increase in prevalence over time, due to a multitude of factors. This is predicted by the International Diabetes Federation, with over 643 million people expected to be living with T2D by 2030. T1D incidence increases annually by 3%, with around 79,000 new cases in children under the age of 15 each year, with this disease epidemiology continually on the rise [3,34,35,36,37]. 

### 3.2. Treatments and Limitations

Currently, exogenous insulin is the only widely available treatment for alleviating the chronic hyperglycaemia and associated complications attributed to T1D. This is indicative of the fact that this autoimmune disease causes a complete failure of the β cells to secrete insulin and thus maintain glucose homeostasis, necessitating the use of exogenous insulin replacement therapy [4,13,38]. In the last 20 years, the use of insulin pumps to deliver continuous short-acting insulin has significantly increased, with this method being able to mimic the human’s natural insulin production, offering many benefits to patients compared to alternative treatments [39]. However, this treatment still requires an exogenous source of insulin. As a result, other potential therapies have been researched, in the aim of discovering a singular treatment to account for all required elements to make it effective. Primarily, this research has included pancreas or islet cell transplantation, which aim to replenish the lost β-cell reserves of the body [20,25,40]. Either way, both treatment modalities, whether it be via insulin or transplantation, possess clinical and practical constraints and limitations [9,41]. Specifically, key limitations are that there is the inability of patients to achieve a consistent and near physiological/homeostatic control of glucose via administered exogenous insulin, or the shortage of suitable pancreatic islet donors, following appropriate screening and donor programs [19,41]. Such limiting factors have recently been at the forefront of efforts to develop renewable sources and protocols for β-cell replacement therapy [40,42].

The focus of current therapies for T2D are treating hyperglycaemia via enhanced insulin production, which may be achieved via drugs such as sulphonylureas, or alternatively enhancing insulin sensitivity via medications such as metformin [43,44]. Continuous overstimulation of the β cells in the pancreas occurs as a result of antidiabetic drug therapy and chronic hyperglycaemia in many T2D patients. Subsequently, tissue exhaustion is observed, resulting in the destruction of the β cells. This destruction will eventually lead to complete lack of insulin production, mimicking T1D. Hence, for T2D patients there is an increasing reliance on insulin therapy [45]. Other limitations to the currently utilised therapies include failure to address the bile acid dysregulation, dysbiosis, and inflammation associated with T2D which perpetuate the hyperglycaemic state and result in further pancreatic cell damage [43,46,47]. T2D complications such as cardiovascular disease, metabolic disturbances and tissue necrosis still remain largely inevitable, with a direct correlation to the aforementioned inflammatory processes and physiological disturbances. This occurs unless the underlying causes of bile acid disturbances, inflammation and dysbiosis, are addressed [48,49]. A review by Negrulj et al., focused on the potential applications of bile acids as a treatment modality in the management of T2D using the novel advanced drug delivery platform of Artificial Cell Microencapsulation (ACM) via the Vibrating Nozzle Method (VNM), and may be of particular interest to the reader [50]. The concept of ACM is described as the development of a semipermeable microcapsule, described in 1964 by Thomas Chang [51,52]. Research has also been carried out by applying this drug delivery technique to the antidiabetic drug Gliclazide with promising in vitro results, and further optimized the delivery platform using the potent antioxidant/anti-inflammatory drug probucol [53,54,55,56]. Interestingly, ACM technology utilizing the VNM is also highly applicable to the effective delivery of pancreatic β cells [57,58,59]. 

A bioartificial pancreas is the current therapy that researchers are investigating as an effective treatment option in both T1D and T2D; this would ideally be capable of supplying a sufficient amount of insulin and will be discussed throughout this review [26,29]. Thus, efforts for the effective delivery of β cells for in vivo applications are highly relevant to not just T1D patients who are the most notable beneficiaries, but also for T2D patients as complete loss of β-cell function is the unfortunate inevitable sequelae of this disorder [1,2,3]. 

## 4. β-Cell Biology and Mechanism of Insulin Release

In diabetes research, an obvious and particular area of interest revolves around islet cell biology, associated with islets of Langerhans. This interest has resulted in highly active research, centred upon insulin-producing β cells, which, theoretically, can restore physiological glucose levels and insulin responses when effectively administered [40,60,61]. There are five different islets of Langerhans endocrine islet cell types that have been discovered, all playing a major role in controlling metabolic fuel homeostasis. β cells are considered the most prominent and contribute to approximately 60% of all islet cells. The remainder of these endocrine cell types include α-cells (30%), and the remaining 10% of human islets consist of somatostatin-secreting cells, the pancreatic polypeptide-secreting cells and ghrelin-secreting cells [60,61]. 

The release of insulin via the islets of Langerhans is in direct proportion to blood glucose levels. Via the glucose transporter 2 (GLUT-2), an increased level of glucose in circulation triggers glucose uptake into pancreatic β cells. Glucose metabolism is essential for glucose-stimulated insulin secretion (GSIS) [60,62]. More recently, it has been postulated that there is a glucose-stimulated biphasic insulin secretion involving two pathways. The most predominant pathway involves the ATP-sensitive potassium channel-dependent pathway. When at rest, the potassium ion channel is open in β cells, and in the absence of glucose, β cells are hyperpolarised. Glucose metabolism closes this channel, which results in depolarisation of β cells and subsequent electrical activity which in turn causes a calcium influx via the voltage dependent calcium channel, increasing the secretion of insulin. The metabolic action of this channel is controlled both by ATP, which inhibits the channel, and magnesium–ADP which stimulates the potassium channel. GSIS is dependent upon the Ca^2+^ ions, hence why the primary cause for the loss of GSIS is considered to be the glucose’s inability to fully depolarise β cells, decreasing all subsequent steps of the pathway and the influx of Ca^2+^ [61,63]. 

## 5. Insulinoma Cell Lines and Their Derivatives

Ideally, the deployed cell system for the effective administration of β cells should incorporate a high insulin content; the cells used should be able to produce sufficient amounts of insulin to meet the body’s requirements. They should also control insulin gene expression and process and release insulin in response to glucose and other physiological stimuli. These cells should be deployed via an advanced delivery system to ensure they are long lasting, durable enough to be clinically feasible, highly sensitive to extracellular glucose levels, biocompatible and nonimmunogenic [64,65]. 

Historically, there have been many cell lines investigated for use as insulin-secreting cells, particularly for the purposes of research and studying the encapsulation materials and capsule matrices using cell lines. Figure 2 provides a schematic of the encapsulation of cell lines such as insulinoma lines. The methods by which these cell lines have been generated are principally via two distinct protocols, either through radiation or virus-induced insulinomas. Some of the first published attempts at generating such cell lines capable of producing insulin were created via implantation of islet cell tumours to the golden hamster [65,66,67]. Although no cell line will perfectly mimic the β-cell physiology evident under homeostatic conditions in a healthy animals or humans, they are, nonetheless, a useful tool in studying molecular events that result in β-cell dysfunction. They also possess the potential to be a source of transplantable tissue in order to overcome the limited availability of primary islets required as part of transplantation, as well as being of use in studying developed microcapsules themselves [65].

The most widely used insulin-secreting cell lines include RIN, HIT, MIN, BRIN, INS-1, NIT and βTC cells which will be discussed in detail below. All these β cells are derived from animal insulinomas, induced via sublethal irradiation or an expressed oncogene [65,67,68]. Glucose transport and phosphorylation via the GSIS will be discussed for the various cell lines concerned. This is an important pathway which is physiologically unique to each cell line and vital for glucose metabolism and insulin secretion [69].

RIN lines proliferate readily and are derived from transplantable islet cell tumours (irradiated) in rats and secrete insulin, somatostatin, and glucagon [65,67,68]. From the initial tumour, two principal cell lines were derived: RINr and RINm. Despite their promise, both cell lines are associated with intrinsic disadvantages. RINm is nonresponsive to glucose stimulation and secretes somatostatin, with its most limiting factor being that insulin levels decline substantially with progressing passages or splitting of cells. Disadvantages of RINr cell lines include the fact that they do not possess the normal properties of glucose transport or phosphorylation, and also do not have the ideal sensitivity to glucose levels. Ideally, the cell lines would respond to glucose at a similar concentration to islets, maintaining GSIS, something neither RINm nor RINr can do, with RINr responding at concentrations <5 mM, and RINm not at all. Following the generation of these two lines, subclones were developed, secreting both peptide products; insulin and somatostatin [65,69]. 

With the aim to discover a cell line with a high rate of insulin secretion, subclones of the RINm line were derived. This attempt resulted in the generation of RIN-m5F from the New England Deaconess Hospital rat strain via high-dosage X-ray irradiation [64,65,66]. The subsequent RIN-m5F cell line exhibited modest GSIS activity, with a 50% increase in insulin release along glucose levels of 0 to 1.4 mM. However, this new cell line still displayed co-secretion of somatostatin and glucagon [64]. As levels of these copeptides are relatively undisturbed in diabetes, it is clinically inappropriate to harvest a bioartificial pancreas which secretes hormones not altered in diabetes [70]. In order to resolve this issue, a subclone of the RINr line was devised, and led to the generation of RINr1046-38 which displayed only insulin secretion and did not secrete the peptides unnecessary for this research [64]. Unfortunately, this cell line also has limitations. Despite being transfected for glucokinase expression and improving upon the parent cell’s insulin content by up to 60-fold, hexokinase had a high native expression which resulted in maximal insulin release, even at very low concentrations of glucose [67].

When studying insulin secretion of various cell lines, a significant limitation is the complete or lack of a response to glucose [69,71]. Thus, in an attempt to overcome this limitation, electrofusion of normal New England Deaconess Hospital rat pancreatic β cells with immortal RIN-m5F was implemented. This resulted in the development of new insulin-secreting cells, with three clones developed in total. These subclones are BRIN-BG5, BRIN-BG6 and BRIN-BD11, BRIN-BG5, with their nomenclature derived from the β-cell and RIN cell fusion. This was conducted with each of the cell lines being cloned from the B well into three wells G5, G7 and D11, hence their respective nomenclature [68,72]. These cell lines have distinct advantages, expressing GLUT-2 and possessing glucokinase activity, however, like RIN cell lines, they express hexokinase, as will be discussed below [65]. 

These hybrid BRIN cells display the ability to transport and metabolize glucose via glucose-sensing mechanisms representative of pancreatic β cells [73]. These clones displayed greater insulin secretion; BRIN-BG5 and BRIN-BG7 respond to glucose at more relevant concentrations, and in contrast to BRIN-BD11 there was a two-to-three-fold increase in insulin output, displaying a response to glucose concentrations of 4.2–16.7 mmol/L [67,72]. Whilst these lines display improved insulin secretion and glucose response in comparison to RIN lines, it is still well below that of pancreatic β cells [67]. In order to further characterize their glucose response and insulin release, several experiments were conducted. One of these tests was for GLUT-2, a membrane-bound glucose transporter protein which plays a role in glucose transport and was examined for its presence in the membranes of these cell types. Results showed its evidence in all three subclones, however, BRIN-BD11 showed the highest expression of GLUT-2, hence suggesting optimal glucose transport mechanisms [65,66]. 

In addition to the aforementioned transporters, enzymes involved in sensing glucose such as hexokinase and glucokinase were compared between BRIN cell lines and normal rat islets, with high β-cell glucose signalling occurring from an increased glucokinase-to-hexokinase ratio. BRIN cell lines demonstrated a much higher glucose phosphorylating level in comparison to the RIN-m5F cell line. Results showed BRIN-BD11 to have the best phosphorylation activity from the three BRIN lines. This, coupled with BRIN-BD11′s high GLUT-2 expression, indicated the greatest glucose sensing ability of the BRIN subclones. Overall, BRIN-BD11 was shown to have retained a near normal β-cell functionality, including the optimal glucose concentration of 16.7 mmol/L required for maximal rat islet stimulation. Novel hybrid β-cell lines (the BRIN family) represent the fusion of ideal properties from each of the original parent cell, expressing both GLUT-2 membrane transporters and containing the glucokinase enzyme with phosphorylation activity. This is in contrast to the RIN-5mF cell line, which lacks this key enzymatic process [68,72]. Therefore, ACM of BRIN, particularly BRIN-BD11, cells seem to be a concept worth further investigation [58,59]. 

Another insulinoma cell line, hamster insulinoma (HIT) cells were generated from preneoplastic islet cells transformed with simian virus 40 (SV40) T-antigen transfection, an oncogenic transfection. This eukaryotic, SV40 viral system is a member of the papovaviral group and has been studied in detail for quite some time. The SV40 virus can also generate the formation of tumours when injected into newborn hamsters and thus has been used in generating the HIT-T15 cell line. HIT-T15 has a modest amount of membrane-bound secretory granules, characteristic of mature β secretory granules in normal hamster β cells, which in comparison with normal hamster islets, is 2.5 to 20 times lower. Despite this, concentration-dependence curves for glucose-stimulated insulin release were similar between HIT-derived cells and normal hamster islets, with peak stimulation (maximal insulin release) occurring at around 7.5–10 mM glucose, which was substantially lower than rat islet cell peaks, being 16.7 mM [65,67,68]. A significant disadvantage of HIT cell lines is their low insulin content, as well as the unresponsiveness to glucose, something common amongst many HIT subclones [18,68]. Therefore, the HIT-T15 cells produced via the subcloning of one original cell line was selected as the final and most advanced subclone of the HIT family due to not only its high insulin content, but also production of glucagon [64].

MIN-6 cells originate from a transgenic C57BL/6 insulinoma mouse, expressing the large T-antigen of SV40 in pancreatic beta cells and has been characterized according to GSIS, glucose transport, glucose phosphorylation and utilization. Impaired GSIS has been attributed to the development and progression of T2D. Notably, MIN-6 cells were developed in an attempt to better understand the cellular mechanisms involved with β-cell dysfunction occurring in diabetes [65,67,68,74]. MIN-6 subsequently demonstrated the ability to secrete insulin at glucose levels up to 25 mmol/L, which is substantially higher than 5 mmol/L glucose occurring under normal physiological conditions [74]. These experimental results assisted in proving that glucose metabolism is a concentration-dependent phenomenon closely related to insulin secretion. The experiments also helped establish that insulin-secreting activity of MIN-6 closely resembled that of normal β cells, making them a useful tool in studying GSIS in normal pancreatic β cells due to this accurate representation at low passage numbers in culture. Unfortunately, similarly to the βTC cell line (discussed below), MIN cells were found to sporadically and unexpectedly lose magnitude in response to rising thresholds of glucose levels required to illicit adequate insulin secretions over time in culture, losing their functionality with increased passages. It is hypothesised that this may be due to an outgrowth of cells with a poor response to glucose or potentially because of declining levels of gene expression responsible for glucose-induced insulin secretion [65,67,68,75,76]. 

A commonly utilized cell line for insulin secretion studies is the insulinoma cell line INS-1 [64]. INS-1 is generated from irradiated cells and predominantly expresses GLUT-2 and glucokinase, thus maintaining glucose levels in the physiological range via appropriate insulin secretion, mimicking normal pancreatic β-cell function, although only 20% of a native cell’s total insulin content is maintained in this cell line [64,65]. Many useful and clinically relevant β-cell features are expressed in this pancreatic cell line, including a high insulin content and insulin-secretory response to glucose within the physiological range (expressed via GLUT-2 and glucokinase) [66]. However, this cell line has a technical disadvantage, requiring the presence of mercaptothion within the culture media, as well as being slow to proliferate. This limits any ACM of this cell line, as mercaptothion is not feasible for appropriate in vivo studies due to its toxicity and ability to denature proteins [64,65]. Subclones have also been developed from this INS-1 line, including the super clone INS-1 832/13. This line was derived from the INS-1 cell line and with the introduction of a human gene, the PR proinsulin gene. The INS-1 832/13 line demonstrates high insulin content in response to glucose, and this has been demonstrated to be sustained throughout continued passages during cell culture. However, similar to the INS-1 cell line, this clone also requires mercaptothion in the culture media [67,68]. 

NIT-1 is another pancreatic β-cell line that was established from the insulinomas developed in transgenic, nonobese, diabetic mice, harbouring a hybrid rate insulin-promoter/SV40 large T-antigen gene [65]. NIT-1 cells have been shown to possess high levels of transcription of insulin, although they also contain the islet hormone glucagon. One distinct disadvantage of this cell line is that it is unresponsive to glucose in the physiological range [67,68]. As with other cell lines from normal differentiated mouse pancreatic β cells, NIT-1 has been shown to possess similar characteristics, including beta granules, extensive Golgi apparatus and well-defined rough endoplasmic reticulum. This is why, despite its limitations, NIT-1 cell lines continue to be used in many studies. NIT-1 cells can not only be utilised in studies for regulation of β-cell function, but also in investigations of T-cell induced β-cell death [77]. 

Similar to the engineering of the MIN cell lines, the beta-tumour cell line (βTC) series was derived from tumours that were generated via transgenic mice expressing the SV40 T antigen under the control of the insulin promoter. This process and cell creation produced immortalised cells that expressed the T-antigens in β cells. βTC cells produce both proinsulin 1 and 2, which the βTC cells can convert into mature insulin [65,67]. Cells secreting insulin from the secretory granules compared to normal β cells have a lower threshold for stimulation, subsequently resulting in the preservation of differentiated β cells without adverse loss of activity for approximately 50 passages in cell culture. One distinct disadvantage of these cells is their expression of hexokinase, which becomes increasingly relevant throughout increased passages, resulting in incorrect insulin secretion for optimal physiological glucose levels, with this being of detriment following around 60 cell passages. However, this has been demonstrated to be downregulated post-growth arrest for a minimum of 2 weeks. Hence, this cell line would be unlikely to be able to be developed for studies of an artificial pancreas [65,68]. 

The first cell lines derived from these transgenic mice included βTC1, βTC2 and βTC3, all with the ability to successfully produce and secrete insulin in response to glucose stimuli, however, the maximum insulin release occurred at 1.25 mM glucose, which is well below the threshold [64]. Through various analysis studies, it became evident that βTC3 showed an increase in insulin secretion and activity along the glucose concentration range of 0.1–16.7 mM. In addition to this cell line, a novel derivative subclone, βTC6 with similar characteristics to βTC3, was derived from transgenic SV40 mice and shown to exhibit a 1.6-fold increase in insulin secretion as a response to glucose stimuli. Staining via immunofluorescence highlighted that cells were heterogeneous, with many secreting only somatostatin, and others glucagon, somatostatin and insulin, regardless of the fact that the expression of the SV40 oncogene was promoter-directed and should, in theory, only allow for insulin gene expression [64,78]. From this βTC6 subclone, another cell line was derived, βTC6-F7, via clonal selection. This line maintains insulin secretion induced via glucose throughout prolonged culture work, and also expresses mRNA for transporter GLUT2 and high glucokinase [67]

In addition to the previously discussed βTC cell lines, various βTC subclones have been established via SV40 transgenic mice into the C3HeB/FeJ strain [78]. One of these newly developed cell lines showed comparable effects to normal cell lines, which included βTC7 [18,64]. Despite the aims of this cell line, as it grew and underwent further passages, the dose–response underwent a left-shift, in correlation with hexokinase expression [79]. This loss of insulin secretion function that occurs with subsequent cell divisions limits the clinical usefulness of such insulinoma cell lines in the development of a biological pancreas for transplantation [80,81,82]. 

## 6. Islet Transplantation

The primary objective for the isolation of islets for either in vivo transplantation or in vitro studies is to obtain viable and purified islets which best represent their function in vivo. Many historical issues with the utilization of fresh islet cells have come from difficulties associated with the heterogeneity of cells and loss of function during prolonged culturing. Key steps pertaining to the successful isolation of islets firstly involves using enzymes that are capable of digesting tissues that connect islets to exocrine tissue, and most commonly, collagenase or trypsin are used. The next steps involve the separation of islets and non-islets, followed by culturing of isolated islets in an environment where cell viability is maintained [25,72,83,84]. 

Isolation of islets from rodent pancreatic tissue can be conducted through various methods, as reported in the literature. From this, two prominent approaches are dependent on when digestive enzymes are added to pancreatic tissues surrounding islets. The first method to enzymatically digest, and therefore, remove the tissue surrounding the islets involves taking the pancreas that has been excised from the euthanized animal, then cutting them into small pieces, approximately 1–2 mm, and placing them in collagenase to digest. In addition to this enzymatic digestion, the pancreatic pieces are, simultaneously, mechanically digested by being shaken or stirred. The second method does not involve cutting the pancreas or mechanical digestion, instead this method involves injecting collagenase into the common bile duct of a euthanized animal, excising the pancreas and digesting it at 37 °C [83].

The ideal transplantation of islets would be in the form of naturally derived primary sources, extracted from healthy animals ready for microencapsulation [85,86]. Most commonly this has historically involved extracted pancreatic islets from rodents and pigs, either in the adult stage or in the form of foetal islet transplants [85,87]. Foetal islet transplants are favoured, as the exocrine pancreas in the adult stage greatly interferes with the function of insulin-producing β cells in vivo [85,88]. Both foetal and neonatal islet tissue has the potential to develop into a functioning endocrine organ when transplanted, and clinical success has been demonstrated in diabetic patients [85,87]. Additionally, foetal islets have a high growth potential, behaving in a similar manner to insulinoma cell lines, with the added advantage of not being cancer cells. Their purity indicates that they can be transplanted without the fear of acinar tissue causing damage [85,88]. 

In 2011, a human insulinoma cell line was created using targeted oncogenesis in human foetal tissue. With the aid of transduction using the lentiviral vector SV40LT under the control of the insulin promoter, transduced buds were grafted into mice and subsequently cultured in vitro to generate the EndoC-βH1 cell line which successfully secreted insulin under glucose stimulation [89,90]. This cell line is stable and retains full functionality at up to 80 passages. The most exciting aspect of this cell line development was that upon transplantation into chemically induced diabetic mice, the diabetes was discovered to be reversed. Upon removal of the transplanted EndoC-βH1 cells, mice developed hyperglycaemia, indicating that the glucose control obtained by the animals was indeed attained from the transplanted cells [90]. Furthermore, the research from Ravassard et al. has been verified independently and further examined, with other sources confirming this EndoC-βH1 cell line can be utilised in screening of novel antidiabetic drugs and is a functional human β-cell line [91]. However, of note from this initial research by Ravassard et al., was the hypoglycaemia that developed after 35 days post-transplantation due to continued expansion and proliferation of the insulinoma cells, necessitating their removal [90]. This limits the use of insulinoma cell lines, due to the fact that primary islets extracted from animals or humans, including foetal pancreatic cells, perform in more physiologically predictable patterns, indicating greater suitability to the clinical scenario of diabetes management [92]. 

The replacement of endocrine tissue in the form of a whole pancreas is rarely performed in current practice due to the complexity of the procedure and the profound side effects of immunosuppressive drug regimens. Complicating the matter further is the very low numbers of healthy organs with worthy, transplantable pancreatic tissue [27,85,93]. As the aim of managing diabetes mellitus is glycaemic control in order to improve quality of life, in contrast to lifesaving transplants such as heart and lung; whole pancreas transplantation is rapidly fading into history as costs and complications do not justify the potential benefits in the small number of recipient patients. It is worth noting that diabetic patients reserve full exocrine pancreatic function even in the most advanced forms of the disease, thus they do not need the entire bulky mass of whole pancreatic organs [93]. Therefore, future transplantation studies should focus on primarily the islets of Langerhans where insulin producing β cells are present; as such this will be discussed in greater detail below [25,85,93].

Human whole-islet transplantation is much preferred to whole pancreas or even isolated, purified β cells, as the process is relatively simple and islets can be quantified and implanted whilst still retaining the glucose-stimulated, complex endocrine functions expected of a complete pancreatic functional subunit [25,85,93]. In terms of functionality, whole islets are much more responsive to GSIS and mimic a fully functioning pancreas more closely than isolated, purified β cells. The rationale for this is that during a hypoglycaemic episode, transplanted isolated, purified insulin-producing β cells cannot suppress insulin release due to loss of complex interconnected signalling pathways that occur within whole islets. This is due to the total loss of cell-to-cell contact that would otherwise occur in aggregated islet cells as part of whole islet subunits [25,85,93,94]. These cell-to-cell contacts are required for the response to fluctuating glucose levels and the subsequent regulation of insulin secretion [95]. 

## 7. Cell Microencapsulation

ACM is a technique that can eliminate or minimize host immune responses to islets via the encapsulation of cells within a biocompatible and semipermeable membrane. The process can be simply defined as the entrapment or envelopment of a predetermined core material, whether it be islet cells, bacteria, or drugs, within a semipermeable membrane using various techniques. The production technique used to prepare the polyelectrolyte-polymer microencapsulation system commonly involves the VNM or a simple extrusion method, with the VNM to be discussed in detail below [96,97,98,99,100,101]. 

Alternative techniques to the VNM may also be considered for use. Such techniques may include spray drying, freeze drying, spray cooling, coating techniques or coacervation methods [102]. Advanced methodologies which may also be implemented include water and oil systems, microfluidic systems, and conformal coating [103,104].

Tomei et al., described such a method of conformal coating encapsulation in detail, with conformal coating described as the coat conforming in a uniform nature and thickness to the size and shape of the materials encapsulated, rather than a constant size coat irrespective of the size of the materials encapsulated, hence, developing capsules of varied sizes for the varied islet sizes, rather than equally sized capsules [103]. Conformal coating aims to avoid limitations of alternative encapsulation techniques, particularly via the thinner coating which results in a shorter diffusion path, which allows easier passage of molecules such as oxygen which may ameliorate islet death caused by hypoxia [105]. The methodology used a water jet flow in oil, with islets added to the inner water phase, creating individual islet coating for an islet/hydrogel precursor solution. A microfluidics system was then used to transition the precursor to the oil phase following conformal coating [103].

Sun et al., described the use of layer-by-layer technology and a transglutaminase-catalysed cross-linking reaction to develop an encapsulation shell. Their process involved the resuspension of cells in anionic gelatine, which were then mixed with transglutaminase in phosphate-buffered saline. The cells were then coated layer-by-layer with gelatine, after which the multilayer coated cells were returned to the transglutaminase for 30 min at 37 °C before being collected via centrifugation and washed. The researchers encapsulated both human cervical carcinoma cell lines and MIN6 cells. Results showed high cellular viability and protection from cytotoxic enzymes. However, the encapsulation process and materials used prevented the cells from undergoing proliferation whilst encapsulated. Although, once released from the capsule, cells resumed the cell cycle and proliferation, indicating that this system may be of use for short-term protection [106].

At its core, cell microencapsulation is the process of encapsulating cells, with the resultant capsules being on the microscale. The fundamental process is promising in the world of cell transplantation, with several benefits including the described reduction in requirements of immunosuppressant medications, the fact that the microcapsules shield the cells from the hosts’ immune system, and that the microcapsule itself can form a functional environment for the survival of the encapsulated cells [107,108,109]. This three-dimensional environment is designed to trap cells within the matrix of the capsule, which must be biocompatible and feature a semipermeable membrane with the correct surface-to-volume ratio to allow for the exchange of nutrients and waste, maintaining the function and viability of the encapsulated cells whilst allowing interactions between cells to occur [110,111,112].

Key to cell microencapsulation is the use of idealistic excipients and polymers in the development of the microcapsules. A wide range of polymers have been suggested for incorporation, with particular focus on the use of sodium alginate. For more information about polymers, both natural and synthetic, used in cell microencapsulation, the reader is invited to consult the following published materials [110,113,114,115].

In 1986, O’shea et al. successfully encapsulated rat islets with alginate and poly-L-lysine, which they implanted into diabetic mice without immunosuppression. The researchers’ xenografts demonstrated a mean survival of 80 days and maintained normoglycaemia for up to 144 days, with nonencapsulated islets having a survival of less than 14 days [116]. This early study highlights the benefits of encapsulation and polymers in the use of islet transplantation. 

Several studies by Soon-Shiong et al. in the 1990s used encapsulated islets with low mannuronic acid alginate with cytokine suppression and high guluronic acid content. The islets were obtained from mongrel placenta and encapsulated. They were transplanted intraperitoneally to diabetic dogs, with encapsulated islets allowing a mean of 105 days without the requirement for exogenous insulin. Control groups who received unencapsulated islets rejected the grafts within seven days. A similar study by Soon-Shiong et al., using alginate of the same characteristics, was encapsulated with islets and injected intraperitoneally to dogs. Results showed maintained euglycemia for up to 172 days without the requirements of endogenous insulin. Furthermore, graft survival was measured via C-peptide release, and found to be maintained for up to 726 days, with results indicating the biocompatibility of the allograft formulation in the absence of immunosuppression [117,118].

One major issue which has long plagued the use of microcapsule transplantations, including those with islets, has been the overgrowth of implants with fibrotic materials from a foreign body response which typically results in reduced or transient islet function from the graft. Bochenek et al., have proposed a method to overcome such issues, via the use of a chemically modified alginate derivative, Z1-Y15. This derivative has been shown as effective in the prevention of pericapsular fibrotic overgrowth. Several materials were used in the development of capsules, including calcium ions for crosslinking and the polymer poly-L-ornithine to mimic capsules used in previous clinical trials, however, the alginate derivative was used in these batches of capsules, in lieu of the standard alginate, with allogenic islets. The capsules were implanted in the bursa omentalis compartment of nonhuman primates to limit movement and clumping. Overall, the results showed viable allogenic islets which were glucose responsive to have survived for four months without the requirement for immunosuppression [119].

## 8. Vibrational Nozzle Method

The VNM uses vibration, causing a breakup of laminar liquid, which, resulting from frequency selection, causes equally sized droplets where the higher the frequency, the smaller the produced microcapsules [50,98,120]. The spherical droplets are formed as a direct result of surface tension [50,120]. The VNM technique also utilises a range of other parameters aside from vibration frequency, including nozzle size, electrode tension and jet flow rate to result in droplet formation [96,98,121,122,123,124]. Homogenous and stable microcapsules may be produced as a result of this novel, effective and promising technique. Such a technique may be deployed via the use of a system such as the Büchi-B390 Encapsulator (Büchi Instruments, Flawil, Switzerland) which is equipped with various nozzle sizes [50,53,54,55,58,59,96]. As with the majority of methodologies, the VNM technique is not without limitations. Limitations include the low production yield due to the fact that only a single droplet forms after another at any given time. The adjustment of the production flow rate may represent a technique of balancing the production yield and the size control of produced microcapsules; increasing vibration frequency or an increase in resultant diameters may assist in such increased production volumes [50,96,120]. 

VNM technology is highly applicable to the encapsulation of labile material such as drugs, as well as bacteria and cells [55,120,125]. The basic theory behind microcapsule formation using this technique is based on the fact that a laminar flow of liquid can be broken up into equal sized droplets if vibrated at an optimal frequency [50]. Liquid droplets are formed as they leave the nozzle system by gravitational forces and surface tension of the liquid from which they emerge but these droplets are not solidified until they reach the gelation or “hardening” bath [42]. This bath most often contains a solution of either CaCl_2_ or BaCl_2_ at various concentrations [30,56,126]. Ca^2+^ has been shown to activate dendritic cell activity in vitro and in vivo and can also adversely affect pancreatic islet cell function within the capsule matrix [127]. On the other hand, leakage of Ba^2+^ ions from alginate microcapsules has been shown to result in accumulation of barium within the bones of rodents, causing weakening of the skeletal system and bone fractures [128]. For these reasons, many authors choose to combine both ions so that lower concentrations of each may be used and added advantages can subsequently be attained by using a dual-divalent ion matrix system [42,93]. 

The mechanism underlying microcapsule formation is ionic gelation, whereby the liquid droplets are collected in the electrolyte bath through a process of ion exchange [50]. More specifically, the mannuronic and guluronic components of alginate (carrying a negative charge) form strong crosslinks with Ca^2+^ by displacing the Ca^2+^ from the Cl^−^ in exchange of Na^+^, and the entire process can be summarized to the theoretical and idealistic level in Equation (1) [129,130,131].

(1) $$SodiumAlginate+Calcium\;Chloride\;\to Calcium\;Alginate+\;Sodium\;Chloride\;2Na\left(Alginate\right)+CaCl_{2}\to Ca\left(Alginate\right)+\;2NaCl\;2\left(C_{6}H_{7}NaO_{6}\right)n\;+\;CaCl_{2}\to \;\left(C_{12}H_{14}CaO_{12}\right)n\;+\;2NaCl$$

The nozzle system utilized in the formation of microcapsules plays a crucial role in the design and layout of the subsequent microcapsules produced. The monocentric nozzle system, often called a simple flow nozzle, is used to encapsulate solid particles, mostly drugs or other biologically active compounds, dispersed within a polymer matrix. This design does not cater for a multilayered microcapsule design and produces beads mostly resembling those produced by the extrusion method, whereby there is a homogenous dispersion of encapsulated contents within the polymer matrix system [120]. 

On the other hand, encapsulation of live organisms such as bacteria for use as beneficial probiotics and pancreatic islet cells involves the creation of a multicompartmental microcapsule capable of providing structural support and rigidity, from a calcium or barium alginate exterior coating. Additionally required is the ability for nutrient and waste exchange in a biologically compatible microenvironment, favouring cell growth, proliferation, metabolic activity, and physiological function, which, in this case, is provided by the alginate–polyelectrolyte membrane [98,132]. The concentric nozzle system may provide a solution to these obstacles as it offers the advantages of forming a multilayered microcapsule, as demonstrated in Figure 1 and Figure 2. This nozzle system, which is compatible with the Büchi-B390 Encapsulator, has two ports (i.e., two nozzles in the one arrangement); one is for the outer shell material consisting of alginate and polyelectrolytes such as poly-l-ornithine; and the other for the inner contents, being, in this example, islet cells in tissue culture media [133]. Scientific theory dictates that the external orifice must be larger than the interior one in order to produce a microcapsule consisting of an appropriate coating and multilayered design. By varying the size of the external nozzle relative to the interior one, microcapsules of varying membrane thickness can be produced. This has important ramifications for the encapsulation of islet cells, as it determines the appropriate membrane thickness required to successfully encapsulate cells and protect them from the outside environment, whilst simultaneously not being too thick and impermeable as to cause hypoxia and cell apoptosis [120,132]. 

Mathematically, the key parameters involved in the formation of droplets using the VNM to encapsulate islet cells are the frequency of the vibration nozzle and the flow rate of the extruded liquid, and an equation can be utilised to illustrate the relationship interconnecting all the parameters. These parameters include the drop diameter (µm) and corresponds to the microcapsule size, the flow rate of the extruded liquid (m^3^/s) and the frequency of the vibration nozzle (1/s) [134]. The equation was derived using a Newtonian fluid mechanics model, as early work using the VNM used Newtonian fluids [135]. However, it should be kept in mind that subsequent work revealed that the model is also applicable to non-Newtonian fluids such as alginate solutions, and has been proven to accurately predict microcapsule size irrespective of the fluid mechanics model [120,134,135]. 

For the microcapsules produced by the concentric nozzle arrangement, the membrane thickness separating the multilayered compartments is important to note, and can also be mathematically represented via another equation. The components of this include the membrane thickness (µm), the diameter of the whole capsule (µm) and solely the core diameter measurement (µm) [120]. 

The extrusion method is also often used in the microencapsulation of islet cells using appropriate polymer-based formulations, however, controlling the droplet size is much more difficult and this method produces beads, rather than microcapsules, a result similar to the VNM method using only the monocentric nozzle setup [93,120,133]. Extrusion-based bead production involves mixing islet cells with the alginate polymer solution, which may contain other excipients such as polyelectrolytes, discussed further below. The resultant mixture is then forced through a syringe nozzle in order to form droplets created by the liquid’s surface tension under the influence of gravity, into the hardening/gelation bath to form solid beads [93,129]. This method produces a uniform dispersion of encapsulated islets within the polymer matrix but cannot form a multilayered design capable of containing cells in a unique ecosystem surrounded by a size-controlled membrane, as seen in Figure 1 and Figure 2 [120]. 

Mathematically, an equation can be used to describe droplet formation using the simple extrusion method. This involves the droplet diameter (µm)_,_ the nozzle diameter (µm), the liquid’s surface tension (µm)_,_ acceleration due to gravity (m/s^2^) and the fluid density (kg/m^3^). There is also a correction factor, which is usually designated as from 0.73 to 0.85 [93,136]. As demonstrated by the parameters in this equation, it is difficult to control the resulting bead size based on the droplet size, as evident by the correction factor, using the extrusion method. 

The extrusion method produces microcapsules of varying and large size, often between 800 and 1000 µm. This is mainly due to the fact that, unlike VNM technology, no defining parameters such as frequency of the jet stream can be applied and altered to produce the desired droplet sizes. In addition, by using simple extrusion methods, it must be noted that the droplet size exiting the nozzle, or in this case from the end of a syringe, does not correspond to the actual bead produced in the gelation bath, and the variation can be anywhere from 5% to 20% [85,120]. This size difference makes this method difficult to implement in terms of islet encapsulation, as islets typically have a diameter of approximately 150 µm, which would be encapsulated in this large microcapsule. Accordingly, to transplant enough for a functional unit, a very large volume would be required in comparison to a 250 µm microcapsule that may result from VNM technology [85]. 

As the flow of the liquid cannot be controlled using frequency, the optimal wavelength of laminar jet liquid breakup cannot be determined and droplets of various sizes are produced, therefore resulting in beads of varying encapsulated contents [85,135]. This is a particularly serious drawback considering the encapsulation of pancreatic islets, particularly in the case of rodent islet cell lines [137]. Variations in the encapsulated cell content can result in inconsistencies in the in vivo response, due to the beads containing variations in both cell activity and function. In addition, islet necrosis can occur if large clumps of islets are encapsulated in beads as opposed to a homogenous dispersion of small and equally sized islets within the core of the microcapsules [137]. VNM technology utilizing the concentric nozzle setup can allow for the constant and well-controlled delivery of islets into the microcapsule core, and also tightly regulate the laminar jet stream breakup into the gelation bath, resulting in homogenous microcapsules with nearly identical core contents surrounded by multilayered membranes of desired thickness and permeability [120]. Figure 2 shows the microcapsules formed via the two main methods deployed in pancreatic islet and β-cell encapsulation. Note that the extrusion method produces microspheres, meaning they have no well-defined boundaries; whilst the VNM method deploying the concentric nozzle setup would produce multicompartmental microcapsules with defined boundaries and layered structural design [58]. 

## 9. Islet Encapsulation and Current Progress

Current developments in the world of cell encapsulation in terms of diabetes mellitus have indicated the potential of stem cell-derived insulin-producing cells; for example, insulin-producing β cells from patient-induced pluripotent stem cells (iPSCs). Patient-derived iPSC β cells have been used for investigations of disease progression and modelling, permitting investigations of potential drug candidates. Encapsulation devices with allogenic stem cell-derived pancreatic progenitors have been proposed, with several advantages, with iPSC islets having the potential to remove the requirement for the patient to be on immunosuppressive medications after transplant. The iPSC islets will allow for insulin-secreting β cells as a personalised cell-replacement therapy [105,138,139].

Patient-derived or specific iPSCs also have the further advantage of a personalised disease model, which, when used in therapy, may overcome the major obstacles in terms of patient immune rejection and mismatches. The use of personalised iPSCs as a disease model also offers enhanced targeted therapeutic modelling for patients, as well as being of use for any personalised pathogenic mechanisms of action [140].

Legøy et al., studied the encapsulation of human iPSCs which were differentiated to β-like cells with alginate, encapsulating single cells. The authors’ in vitro results indicated that the encapsulation of individual cells allowed oxygenation, with no hypoxia indications, in contrast to results of islet aggregates. Alginate encapsulation also permitted improved differentiation of the human iPSCs, with their proteome resembling an islet-like signature [141]. The same authors also investigated human iPSCs derived from pancreatic progenitors which were also encapsulated with alginate and underwent xenotransplant to normoglycaemic mice for 2 weeks. Their results indicated that the in vivo exposure of the encapsulated iPSCs resulted in increased expressions of the islet-specific hormones, somatostatin, glucagon, and, importantly, insulin, with islet proteome characteristics improved [142].

Stock et al., developed microcapsules of stem cell-derived islets, manufactured via a microfluidic system with conformal coating, with the detailed method described previously. Their conformal coated stem cell islets were able to secrete physiological levels of insulin, and when implanted in NOD-scid mice, they acted in a similar manner to unencapsulated stem cells and maintained euglycemia at human levels for more than 80 days [105].

In terms of clinical trials, several are currently ongoing, including one currently investigating the βAir (BetaO2 Technologies Ltd., Israel) device (NCT02064309). This device is a macroencapsulation system of human pancreatic islets and alginate. Initial results from the trial published in 2018 demonstrated the device to be safe, showing that despite the absence of systemic immunosuppression, no subjects showed donor-specific HLA antibodies. The transplanted β cells within the device survived the trial, with the trial overcoming the aforementioned issues of oxygenation associated with macroencapsulation via the incorporation of a refillable oxygen tank, which must be refilled daily via subcutaneous Port-A-Cath ports. Despite the β-cell survival, no impact on metabolic control was observed, with only minute levels of C-peptide able to be detected. Hence, the results showed a safe device, allowing the survival of β cells, but limited functionality of the transplanted β cells [143]. 

Several trials are also investigating the use of stem cell-derived islets. One current trial by Vertex Pharmaceuticals is underway, (NCT04786262). The trial is investigating their product VTX-880 in T1D. The VTX-880 is a differentiated human stem cell-derived pancreatic islet therapy. The Phase I/II clinical trial aims to investigate the safety, tolerability, and efficacy of the infusion of VTX-880. The trial infusion also involves the coadministration of immunosuppressive therapies to prevent the rejection of the infused islet therapy. The potential treatment of VTX-880 would restore pancreatic islet cell function, restoring the body’s glucose levels [144]. 

A trial by ViaCyte (NCT04678557) is in a clinical phase, in collaboration with the material science company Gore and is currently in Phase II for a therapy for T1D via an encapsulated cell therapy. Their VC01-103 cell therapy candidate will be implanted subcutaneously in patients as part of the trial [145]. A previous clinical trial by ViaCyte (NCT03163511) investigated their VC-02 product. The study implanted macro delivery devices containing pluripotent stem cell-derived pancreatic endoderm cells subcutaneously. Patients were also given pharmacological immunosuppression as the device does not offer immune protection. The implanted devices resulted in measurable insulin release, and peripheral blood samples showed C-peptide levels to be meal-responsive in the subset of subjects. Additionally indicated was the lack of autoimmune response to the device, highlighting the likely effectiveness of the immunosuppressive medication in the patient subset [146].

## 10. Limitations

The main limitation to insulinoma-derived cell lines is the varying insulin-secretory responses to glucose when compared to normal β cells [65]. Unfortunately, many cell lines have unstable phenotypes which tend to dedifferentiate with time in culture, resulting in a decrease in insulin content and GSIS, as well as a left shift in the glucose dose–response curve [64]. In other terms, cell lines have a disproportionate release of insulin in response to glucose the longer they remain in culture; whereby the predicable and rational insulin release in response to varying glucose concentrations as expected under physiological conditions becomes difficult over time. For instance, HIT and βTC cells secrete insulin in response to glucose with higher sensitivity based upon concentration-dependence curves, while BRIN, MIN 6 and INS-1, retain their normal glucose regulation of insulin release [65].

### 10.1. Pharmaceutical Drawbacks of Microencapsulation

Microencapsulation as a delivery platform for transplanting pancreatic islets and β cells does encompass inherent drawbacks and limitations. Biocompatibility is by far the biggest factor to consider, and this can be due to the membrane system (alginate and/or other excipients) or the encapsulated cells themselves, as previously noted [147]. Purification of alginate will improve the biocompatibility profiles, as alginate does not allow for impurities such as proteins, endotoxins as well as heavy metals, as they are all potentially detrimental to the encapsulated contents, in addition to being able to activate the immune system [148]. Controlling the size of the microcapsules is important, as smaller sizes are less likely to activate cellular immune pathways, are easier to transplant, and oxygen perfusion and exchange of nutrients/waste products is greater. However, they have the drawback of containing far less cells, so are, therefore, clinically less valuable [147,149]. Poor mechanical stability can also lead to microcapsule breakdown and release of contents, which in turn would lead to an overt immune response and rapid graft loss [147,150]. From a manufacturing point of view, microencapsulation will need to be drastically scaled up if realistic clinical benefits are to be attained, as current protocols are somewhat limited to small scale research studies and not apt for rapid widespread production [120]. Indeed, a range of hydrogel-bile acid-polyelectrolyte formulations are being validated for future applications [151,152,153,154,155,156,157,158,159,160,161]. 

### 10.2. In Vivo Limitations

Upon implantation, islet-containing microcapsules may have unpredictable or less than satisfactory outcomes. Such results typically occur due to immune system destruction of the device, and hypoxia (from poor vasculature), cell necrosis (due to high packing density and/or hypoxia) and poor insulin kinetics have become a major concern [85,92,150]. Microencapsulated islets do not readily respond to fluctuating glucose levels, as insulin diffusion through the multiple layers of the delivery membrane system can be delayed, low or unpredictable. Timely release during mealtimes (bolus release) and reducing outputs during resting periods (basal) can be problematic. In addition, microcapsules can become clumped or even be displaced at the implantation site, which will further adversely affect insulin kinetics [92]. Adequate vascularisation for oxygen/nutrient diffusion and waste exchange at the site is also a limiting factor influencing islet survival and activity, and the ideal site is yet to be determined due to the fact that this differs for animal studies versus human trials [86,93,95,150]. 

## 11. Future Perspectives

Alginate-based microcapsules are fast becoming the most preferred form of delivery platform for the effective administration of pancreatic islet cells. They have the advantage of not requiring immunosuppressive drugs, organ donation constraints or the feasibility hurdles created by islet cell transplantation [148]. 

The ideal formulatory approach involves the use of polyelectrolytes and synthetic hydrogels to complement the alginate system, resulting in the creation of microcapsules with more biocompatible properties and with the membrane support required for the exchange of nutrients and waste products. However, the “ideal” microcapsule consisting of all aforementioned properties remains elusive, as current drawbacks of hypoxia, immune system activation, fibrotic overgrowth and poor/low clinical response and low production yield continue to create significant challenges [42]. 

Several approaches have been developed in order to optimize the microencapsulation of islet cells for the generation of a successful bioartificial pancreas. Pretreating islet cells with desferrioxamine reduced hypoxia, and coating alginate microcapsules with heparin reduced the fibrotic overgrowth occurring in vivo, as studies have shown. Additionally, biological factors such as glucagon-like-peptide 1, hepatocyte growth factor and vascular endothelial growth factor can be coformulated with islet cells to stimulate their function, making them more biologically active [95]. Bile acids have been coformulated with hydrogels and polyelectrolytes and have demonstrated the ability to enhance encapsulated pancreatic β-cell viability and function and result in the creation of more mechanically stable, robust microcapsules with enhanced physicochemical and biological characteristics, but the results have had mixed success, depending on the hydrogel–polyelectrolyte system deployed [58,59].

In conclusion, microencapsulation of pancreatic islets and β cells remains a novel and highly promising technique used to generate a bioartificial pancreas, but some challenges still remain before they can be deemed fully practical and clinically beneficial on a large scale. The progress thus far has been substantial at a pharmaceutical level, but future clinical trials should illicit the practicality and long-term efficacy of such delivery platforms. 

## Data Availability

Not applicable.
